# Phosphoproteomic Analysis of the Highly-Metastatic Hepatocellular Carcinoma Cell Line, MHCC97-H

**DOI:** 10.3390/ijms16024209

**Published:** 2015-02-16

**Authors:** Miaomiao Tian, Han Cheng, Zhiqiang Wang, Na Su, Zexian Liu, Changqing Sun, Bei Zhen, Xuechuan Hong, Yu Xue, Ping Xu

**Affiliations:** 1 State Key Laboratory of Proteomics, Beijing Proteome Research Center, National Engineering Research Center for Protein Drugs, National Center for Protein Sciences, Beijing Institute of Radiation Medicine, Beijing 102206, China; E-Mails: mmtian@genetics.ac.cn (M.T.); wangzq@whu.edu.cn (Z.W.); sunamask@gmail.com (N.S.); 2 Department of Biomedical Engineering, College of Life Science and Technology, Huazhong University of Science and Technology, Wuhan 430074, China; E-Mails: chenghan@hust.edu.cn (H.C.); lzx@hust.edu.cn (Z.L.), xueyu@hust.edu.cn (Y.X.); 3 Key Laboratory of Combinatorial Biosynthesis and Drug Discovery (Wuhan University), Ministry of Education, and Wuhan University School of Pharmaceutical Sciences, Wuhan 430072, China; E-Mail: xhy78@whu.edu.cn; 4 Tianjin Baodi Hospital, Tianjin 301800, China; E-Mail: husband88@163.com

**Keywords:** phosphorylation, phosphoproteomics, metastasis, HCC, spliceosome

## Abstract

Invasion and metastasis of hepatocellular carcinoma (HCC) is a major cause for lethal liver cancer. Signaling pathways associated with cancer progression are frequently reconfigured by aberrant phosphorylation of key proteins. To capture the key phosphorylation events in HCC metastasis, we established a methodology by an off-line high-pH HPLC separation strategy combined with multi-step IMAC and LC–MS/MS to study the phosphoproteome of a metastatic HCC cell line, MHCC97-H (high metastasis). In total, 6593 phosphopeptides with 6420 phosphorylation sites (p-sites) of 2930 phosphoproteins were identified. Statistical analysis of gene ontology (GO) categories for the identified phosphoproteins showed that several of the biological processes, such as transcriptional regulation, mRNA processing and RNA splicing, were over-represented. Further analysis of Kyoto Encyclopedia of Genes and Genomes (KEGG) annotations demonstrated that phosphoproteins in multiple pathways, such as spliceosome, the insulin signaling pathway and the cell cycle, were significantly enriched. In particular, we compared our dataset with a previously published phosphoproteome in a normal liver sample, and the results revealed that a number of proteins in the spliceosome pathway, such as U2 small nuclear RNA Auxiliary Factor 2 (U2AF2), Eukaryotic Initiation Factor 4A-III (EIF4A3), Cell Division Cycle 5-Like (CDC5L) and Survival Motor Neuron Domain Containing 1 (SMNDC1), were exclusively identified as phosphoproteins only in the MHCC97-H cell line. These results indicated that the phosphorylation of spliceosome proteins may participate in the metastasis of HCC by regulating mRNA processing and RNA splicing.

## 1. Introduction

Human hepatocellular carcinoma (HCC) is one of the most lethal diseases around the world [1]. One major reason for the high death ratio of HCC is due to the fast invasion and metastasis ability of tumor cells in primary liver cancers. Exploring the mechanism of HCC metastasis can help to develop novel reagents and drugs for the diagnosis or therapy of liver cancer.

In recent years, the development of proteomics has promoted us to detect the dynamic change of the whole proteome in cancer samples easily. However, post-translational modifications (PTMs) endow protein with new functions to respond to different pathological processes in cancers. Phosphorylation is one of the major types of PTMs in eukaryotic species. Phosphorylation or dephosphorylation usually activates and/or inactivates the transduction of many signal pathways, such as the MAPK/ERK(mitogen-activated protein kinases/extracellular signal-regulated kinase) pathway [2], the cAMP (cyclic AMP)-dependent pathway [3] and the IP3/DAG (Inositol 1,4,5-triphosphate/1,2-Diacylglycerol) pathway [4]. Dysfunction of these pathways and mutations of important kinases are always associated with various diseases, including cancers [5,6,7,8]. Based on various phosphopeptides enrichment technologies, phosphoproteomics and quantitative phosphoproteomics can identify aberrant kinase signaling pathways and mutant phosphorylation sites (p-sites) in cancer samples, which helps to dissect the mechanisms of cancer progression.

Moreover, suitable experimental samples should be used for exploring the mechanism of metastasis of HCC. Although several labs had established human and animal HCC cell lines [9,10], only a few of them were proper for research on human HCC metastasis. For example, Li *et al.* had established HCC cell lines with different metastatic potentials, MHCC97-L (low metastasis) and MHCC97-H (high metastasis), from the metastatic hepatocellular carcinoma cell line, MHCC97 [11]. Additionally, these cell lines have been widely used for studying the mechanism of metastasis in HCC [12,13,14].

In this work, we studied the phosphoproteome of a metastasis HCC cell line, MHCC97-H, by an off-line high-pH HPLC separation strategy combined with a multi-step IMAC method. In total, 2930 phosphoproteins containing 6593 phosphopeptides with 6420 p-sites were identified from only 2.5 mg cell lysates. Bioinformatic analysis showed that proteins involved in a number of biological processes, such as transcriptional regulation, mRNA processing and RNA splicing, and pathways, such as spliceosome, the insulin signaling pathway and the cell cycle, were highly phosphorylated in the MHCC97-H cell line. Particularly, compared with a previously published phosphoproteome of human normal livers, U2 small nuclear RNA Auxiliary Factor 2 (U2AF2), Eukaryotic Initiation Factor 4A-III (EIF4A3), Cell Division Cycle 5-Like (CDC5L) and Survival Motor Neuron Domain Containing 1 (SMNDC1) proteins, which are associated with the spliceosome pathway were the unique phosphoproteins identified in the MHCC97-H cell line. Moreover, the function of U2AF2, EIF4A3, CDC5L and SMNDC1 proteins involved in HCC metastasis was rarely recorded. Taken together, the phosphopeptide enrichment strategy established in this study could provide novel phosphoproteins involved in liver cancer.

## 2. Results and Discussion

### 2.1. Phosphopeptides in MHCC97-H by Off-Line High-pH HPLC Separation with Multi-Step IMAC (Immobilized Metal ion Affinity Chromatography)

**Figure 1:**
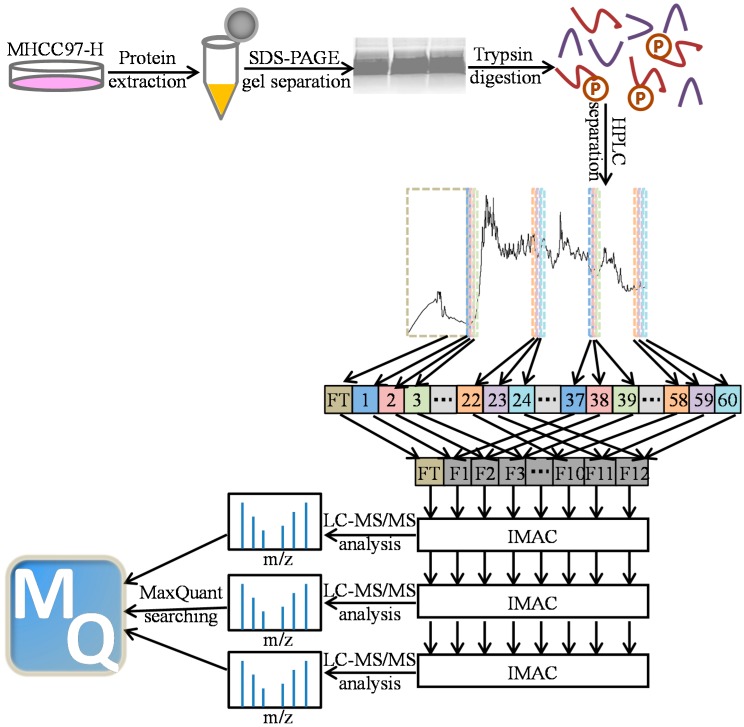
Scheme for sample preparation, HPLC multi-IMAC (Immobilized Metal ion Affinity Chromatography) methods and data processing of MHCC97-H (high metastasis) phosphoproteomics.

**Figure 2:**
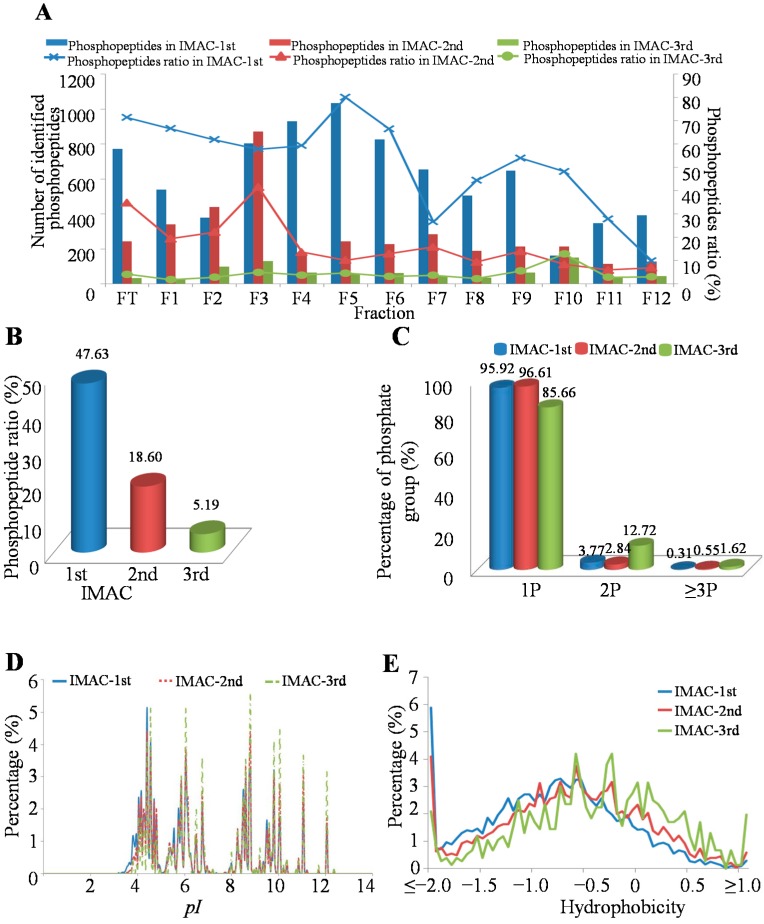
Characteristics of the identified unique phosphopeptides in different HPLC fractions and IMAC steps. (**A**) Distribution of identified unique phosphopeptides in different HPLC fractions; (**B**) Distribution of identified unique phosphopeptides in different IMAC steps; (**C**) Distribution of phosphorylated peptides based on their phosphorylation sites (p-sites) in different IMAC steps; (**D**) The *pI* value distribution of the identified phosphopeptides in different IMAC steps; (**E**) The hydrophobicity distribution of the identified phosphopeptides in different IMAC steps.

In order to globally profile the phosphoproteome involved in liver cancer invasion and metastasis, we performed a systematic phosphorylation profiling with an off-line high-pH HPLC separation strategy combined with a multi-step IMAC (Immobilized Metal ion Affinity Chromatography) method for a human metastatic HCC cell line, MHCC97-H, from 2.5 mg cell lysates (Figure 1). Through this method, we successfully identified 6451, 3279 and 802 unique phosphopeptides in the first, second and third round of IMAC steps, respectively (Table S1). In order to systematically investigate this method, we firstly compared the purity of enriched phosphopeptide samples from different fractions and different IMAC steps with the enrichment ratio as the index. The result indicated that the distribution of the phosphopeptide ratio was uneven in those conditions. For example, the highest phosphopeptide enrichment ratio was in Fraction 5 (80.05%), while the lowest phosphopeptide enrichment ratio was in Fraction 12 (9.94%) when performed in the first round of IMAC enrichment. In the second round of IMAC enrichment, the highest phosphopeptide ratio existed in Fraction 3 (41.61%), while the lowest phosphopeptide ratio existed in Fraction 11 (6.04%). However, in the third round of IMAC enrichment, the highest phosphopeptide ratio was found in Fraction 10 (12.94%), while the lowest phosphopeptide ratio was found in Fraction 1 (1.86%) (Figure 2A). We also analyzed the phosphopeptide enrichment ratio in different fractions, and the results showed that the highest phosphopeptide enrichment ratio was found in Fraction FT (Flow Through) (36.81%), while the lowest phosphopeptide ratio existed in Fraction 12 (6.67%) (Figure 2A). This result indicated that a large proportion of phosphopeptides existed in the FT fraction when the peptides were separated by the high-pH HPLC strategy. However, the phosphopeptides ratio was decreased by more IMAC steps; for example, nearly half of all phosphopeptides (47.63%) were identified in first IMAC step, 18.60% in the second IMAC step and 5.19% in the third IMAC step (Figure 2B). The reason for the observation may be derived from the protein sample being limited in this study. Additionally, more starting cell lysates will be used for further phosphoproteomic study in our lab.

Furthermore, we detected the p-sites of identified phosphopeptides in our method. The results showed that almost all of the identified phosphopeptides contained one p-site either in the first round of IMAC enrichment or the third round (Figure 2C). However, increasing the IMAC steps was beneficial for improving the ratio of phosphopeptides with multi-phosphorylation sites (14.34%) in one experiment. This result suggested that more rounds of the IMAC enrichment strategy can help us to identify complicated phosphopeptides with multiple p-sites.

In order to investigate the chemical characteristics of phosphopeptides in different rounds of IMAC steps, we firstly compared the pH values of the identified phosphopeptides in three rounds of IMAC enrichment. The results showed that most phosphopeptides were acidic phosphopeptides with low *pI* values in these three rounds of IMAC steps. However, the ratio of acidic phosphopeptides was decreased by more rounds of IMAC enrichment (Figure 2D). We also detected the hydrophobicity of the identified phosphopeptides in these three rounds of IMAC steps. The result showed that almost all of the identified phosphopeptides were hydrophilic peptides in all IMAC steps, while the ratio of hydrophobic phosphopeptides was increased by more rounds of IMAC enrichment (Figure 2E). These results indicated that more rounds of IMAC steps may be helpful for basic phosphopeptides and hydrophobic phosphopeptide enrichment under the high-pH HPLC separation condition.

### 2.2. Characteristics of Identified Unique Phosphorylation Events in the MHCC97-H Cell Line

**Figure 3:**
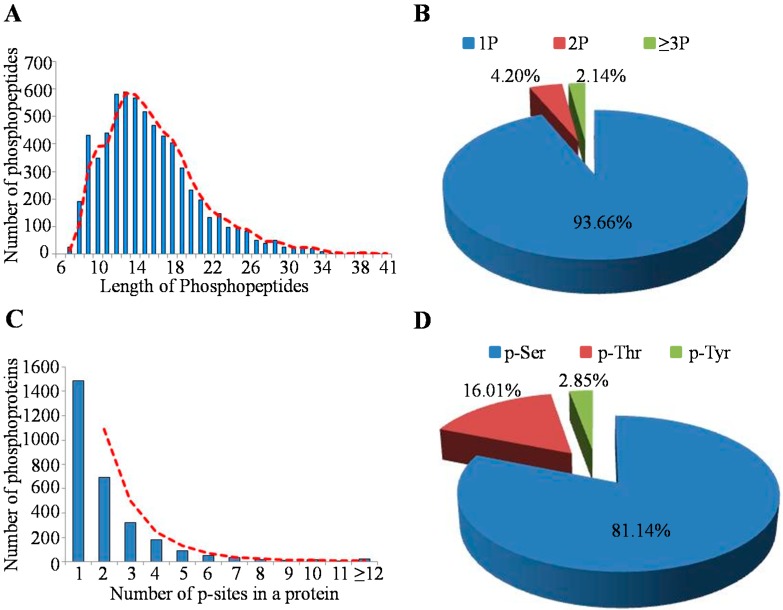
Characteristics of the identified unique phosphopeptides in the MHCC97-H cell line. (**A**) Distribution of phosphopeptides based on their length; (**B**) Distribution of phosphopeptides depending on their number of p-sites; (**C**) Distribution of phosphorylation proteins based on their number of p-sites; (**D**) Distribution of phosphorylation serine (p-Ser), phosphorylation threonine (p-Thr) and phosphorylation tyrosine (p-Tyr) sites in the MHCC97-H cell line protein.

In total, there were 6593 phosphopeptides with 6420 p-sites identified from the 2.5 mg MHCC97-H cell lysate. Depending on their length, these phosphopeptides showed different abundances (Figure 3A); for example, the peptides with lengths of 12, 13 and 14 had percentages of 8.80% (580/6593), 8.95% (590/6593) and 8.60% (567/6593), respectively. Among these identified peptides, most of them were singly-(93.66% (6175/6593)) or doubly-(4.20% (277/6593)) phosphorylated, but there were also 141 peptides that contained at least three p-sites (Figure 3B). The 6420 p-sites occurred on 2930 phosphorylation proteins, among which, approximately 50.78% (1488/2930) owned a single p-site, 23.69% (694/2930) carried two identified p-sites and 10.96% (321/2930) possessed three p-sites (Figure 3C). Generally, the average number of p-sites in a protein was 2.19 (6420/2930). Obviously, the phosphorylated serine (p-Ser) occupied a large proportion of about 81.14% of the identified p-sites, while the percent of the phosphorylated tyrosine (p-Tyr) was relatively less, which demonstrated that the phosphorylation event occurred in serine more often than in threonine or in tyrosine (Figure 3D).

### 2.3. The Preferences for Sequence and Structure Features of Phosphorylation Sites (p-Sites) in the MHCC97-H Cell Line

**Figure 4:**
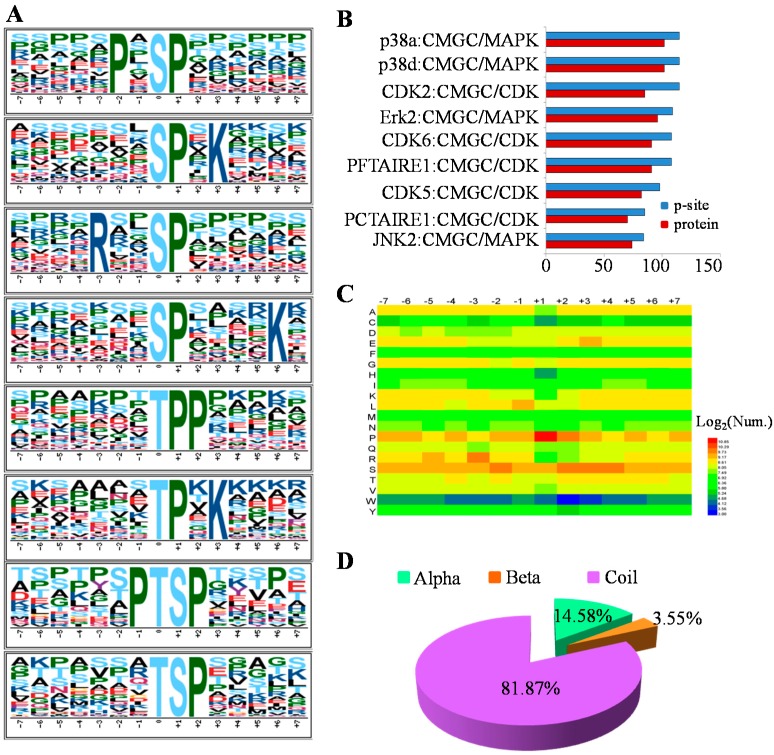
Analysis of p-sites by sequence motif, Group-based Prediction System (GPS) algorithm with the interaction filter, or *in vivo* GPS (iGPS), the distribution of amino acid flanking and structural preferences in the MHCC97-H cell line. (**A**) The sequence motif analysis of p-sites in the MHCC97-H cell line consisting of 14 residues surrounding the targeted site by Motif-X; (**B**) The top 10 protein kinases with the most p-sites by the prediction of iGPS; (**C**) The heatmap for the distribution of amino acids flanking p-sites in the MHCC97-H phosphoproteome; (**D**) The secondary structural distribution for the p-sites.

Phosphorylation sites were considered as the footprints of kinase activities. Therefore, Motif-x [15], an iterative strategy to extract overrepresented patterns by comparison to a dynamic background, was adopted to search for the putative phosphorylation motifs from our phosphorylation dataset. In total, 36 serine motifs and 11 threonine motifs were significantly enriched in the MHCC97-H phosphoproteome (Table S2), and the top four most representative motifs for p-Ser and phosphorylation threonine (p-Thr) are presented in Figure 4A. The known motifs, such as P-*X*-[ST]-P, [ST]-P, [ST]-P-*X*-K (*X* indicates any amino acid residue), could be targeted for mitogen-activated protein kinases (MAPKs) and cyclin-dependent kinases (CDKs) [16]. Acidic motifs, for example, [S]-D-*X*-E, [S]-E-*X*-E, and [S]-*X*-D, might be recognized by casein kinase II [17,18]. Basic motifs, such as R-*X*-*X*-[S], R-*X*-*X*-[S]-*X*-S, R-R-*X*-[S] and R-S-*X*-[S], were potential substrates of Ca^2+^/calmodulin-dependent protein kinases (CaMKs) [18,19]. The results may indicate that these kinases might play crucial roles in the MHCC97-H cell line.

In order to reveal the corresponding kinases of p-sites, Group-based Prediction System (GPS) algorithm with the interaction filter, or *in vivo* GPS (iGPS) 1.0 [20], which was developed for the prediction of site-specific kinase-substrate relations (ssKSRs) in five organisms, was chosen to predict the upstream regulatory kinases of 6420 identified p-sites. In total, 5346 ssKSRs between the 378 kinases and 331 substrates for the 412 p-sites were predicted (Table S3), while the top 10 protein kinases with the most p-sites were picked out and presented in Figure 4B. Obviously, all of the ten kinases belonged to the group named after member families CDK, MAPK, GSK3 and CLK (CMGC), including p38a, p38d, CDK2, and so on, which were also in accordance with the motif analysis that the [ST]-P motif was highly enriched. The kinases in the CMGC group were involved in critical cellular processes: for example, p38s in CMGC/MAPK were highly associated with many signal pathways, such as inflammation, apoptosis and development [21]; CMGC/MAPK/c-Jun *N*-terminal kinase (JNK) regulated embryonic morphogenesis, cell proliferation and apoptosis [22]; the kinases of the CMGC/CDK family were implicated in the cell cycle or cell division [23]. Generally, the high implication of the CMGC group in the MHCC97-H cell line was consistent with the previous analyses that CMGC kinases played a predominant role in the regulation of cellular phosphorylation [20].

Moreover, to reflect the relative abundances of the specific amino acids surrounding the modified site, the heatmap for the items of PSP (7, 7), a phosphopeptide with a length of 15 with seven upstream and seven downstream residues around the p-site, was visualized by HemI [24] in Figure 4C. It is obvious that the P at the +1 position was the amino acid with the highest frequency, and the R at the −2 position was also significantly enriched. Indeed, these results visualized by the heatmap are in agreement with the motif analyses above.

Besides, we also analyzed the secondary structural preferences for the p-sites. By NetSurfP ver.1.1 software [25], the results indicated that protein phosphorylation predominantly occurred in amino acids with a secondary structure of coiled-coil, which had a coverage of approximately 81.87% in this study (Figure 4D).

### 2.4. Functional Analysis of the MHCC97-H Phosphoproteomic Data

**Figure 5:**
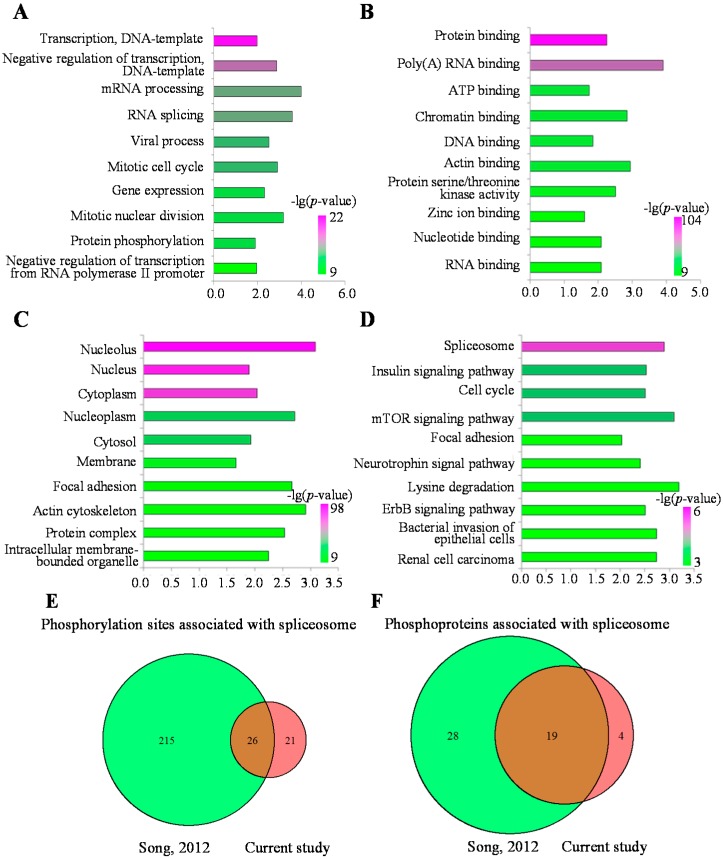
Gene Ontology (GO) annotation and the Kyoto Encyclopedia of Genes and Genomes (KEGG) pathway (top 10) analysis for phosphoproteins. (**A**) Biological process of GO annotation (top 10); (**B**) Molecular function of GO annotation (top 10); (**C**) Cellular component of GO annotation (top 10); (**D**) The most over-represented KEGG pathways; (**E**) Comparison of p-sites associated with the spliceosome between MHCC97-H and the normal liver sample; (**F**) Comparison of phosphoproteins associated with the spliceosome between MHCC97-H and the normal liver sample.

To understand the biological function of cellular phosphorylation in the MHCC97-H cell line, we annotated these phosphoproteins by the enrichment analysis in the Gene Ontology (GO) function term and the Kyoto Encyclopedia of Genes and Genomes (KEGG) pathway. Among the Gene Ontology (GO) annotations, most of the significantly enriched biological processes were associated with the DNA template and transcription processes (Figure 5A, Table S4), which indicated that these phosphoproteins mainly participated in the regulation of transcription from the DNA template. Most significantly enriched cellular components were associated with the nucleus, which indicated that the identified phosphorylated proteins mainly had localizations in the nucleus (Figure 5C, Table S4). Actually, most significant molecular functions were involved in the regulation of the mRNA transcription (Figure 5B, Table S4). Based on the enrichment analysis on the three categories of the GO term, we supposed that the identified phosphoproteins mainly participated in the processes associated with the regulation of the transcription in the nucleus. The details of the enriched terms are shown in Table S4.

To better understand the pathways regulated by these phosphorylated proteins, the enrichment analysis of the KEGG pathways was also applied. The most significantly enriched pathways were the spliceosome, the insulin signaling pathway and the cell cycle (Figure 5D, Table S5). The spliceosome pathway is necessary to help the maturation of the mRNA. The standard spliceosome is made up of five small nuclear ribonucleoproteins (snRNPs), U1, U2, U4, U5 and U6 snRNPs, and several spliceosome-associated proteins (SAPs) [26]. In cancer, it was reported that the alternative splicing processes can be regulated by phosphorylation [27]. To further analyze the mechanisms of spliceosome regulation mediated by the phosphorylated proteins in the HCC cell line, we compared our results with another phosphoproteome profiled in the normal liver tissue sample by Song *et al.* [20]. It was observed that 47 phosphorylation sites were detected in MHCC97-H, among which 26 p-sites were also identified in Song’s work (Figure 5E). As for the phosphorylated proteins, 23 proteins were identified in MHCC97-H, among which 19 proteins were also reported by Song *et al.* (Figure 5F). The other four proteins, including U2AF2, EIF4A3, CDC5L and SMNDC1, were unique phosphorylated proteins identified in the MHCC97-H cell line. Until now, the functions of these four proteins were not clear in HCC, but they participated in the metastasis of other cancers. U2AF2 (also known as U2AF65) is a non-snRNP protein, which cooperated with U2AF35 to help U2 snRNP binding to the pre-mRNA 3' splice site [28]. This protein was reported to participate in breast cancer and the metastasis of cutaneous melanoma through regulation of the spliceosome [29,30]. EIF4A3 is a core component of the splicing-dependent multiprotein exon junction complex (EJC) deposited at splice junctions on mRNAs [31]. A proteomics study showed that EIF4A3 can be used as a serum marker for pancreatic cancer diagnosis [32]. CDC5L is a DNA-binding protein regulating the G2-M transition of the cell cycle. Additionally, a complex with precursor RNA processing 19 (PRP19) is also formed to activate pre-mRNA splicing [33]. Moreover, Sanidas *et al.*, revealed Akt isoform-specific signals linking RNA processing to lung cancer by a phosphoproteomics screening [34]. Dapat *et al.*, also showed that the pathway of RNA processing was most enrichment in human lung epithelial (A549) cells after influenza virus infection by quantitative phosphoproteomics [35]. These research works indicated that the pathway of the spliceosome plays an important role in cancers. Although different pathways or key proteins had been identified to be involved in the development and metastasis of liver cancers by some HCC cell lines, there are few studies showing that the phosphoproteins of the spliceosome pathway participated in the metastasis of HCC. For example, plectin-1(phopho-Ser-4253) and alpha-HS-glycoprotein had been identified as phosphoproteins, which serve as biomarkers for the hepatocellular carcinoma specimen by quantitative analysis of phosphopeptides in stable isotope labeling with amino acids in cell culture for HepG2 cells [36]. Li *et al.*, performed an analysis of the tyrosine-phosphorylated proteins in Hep3B and MHCC97H cell lines and found that (fps/fes related) tyrosine kinase (FER) might serve as a novel drug target for HCC therapy [37]. Quantitative reverse transcription-PCR, Western blotting and immunohistochemistry indicated that CDC5L is the most likely candidate oncogene for the 6p12-p21 amplicon in osteosarcoma [38]. SMNDC1 is a survival motor neuron protein, and it is necessary for spliceosome assembly [39]. Recently, comparative proteomic analyses revealed that SMNDC1 was a key node to regulate the tumor growth and metastasis in ovarian cancer [40]. All of these results indicated that the phosphorylated proteins were associated with metastasis of the MHCC97-H cell line through regulating the spliceosome pathway in the nucleolus.

## 3. Experimental Section

### 3.1. Cell Culture

MHCC97-H, a hepatocellular carcinoma (HCC) cell line, was cultured in DMEM (Dulbecco’s Modified Eagle Medium) medium supplemented with 10% dialyzed FBS (fetal bovine serum) in a humidified atmosphere with 5% CO_2_.

### 3.2. Protein Extraction and Digestion

Cells were harvested and extracted with lysis buffer (50 mM Tris–HCl (pH 8.0), 150 mM NaCl, 1 mM EDTA, 5 mM Na_4_P_2_O_7_, 100 mM NaH_2_PO_4_, 1 mM NaF, 1 mM Na_3_VO_4_, 1 mM C_3_H_7_CaO_6_P, 0.5% NP-40, 1% phosphatase inhibitor cocktail 2 (Sigma, St. Louis, MO USA), 1% phosphatase inhibitor cocktail 3 (Sigma), 1 tablet of EDTA-free protease inhibitor cocktail (Roche, Basel, Switzerland) for every 10 mL of lysis buffer, 1 mM PMSF, 5 mM IAA). The cell lysate was sonicated for 40 s followed by a 20-s rest interval for 10 min. The mixture was centrifuged at 13,000 rpm for 10 min to remove cell debris. Protein was resolved on a 10% SDS-PAGE gel and running for a 0.8 cm length and then stained with Coomassie Blue G-250. The entire gel lane was sliced into 1 mm^3^ pieces followed by destaining and in-gel digestion with 10 ng/μL Trypsin (Promega, Madison, WI, USA,) at 37 °C incubation overnight as per the manufacturer’s protocol.

### 3.3. Peptide Desalting

The resulting peptide mixtures were dried and reconstituted in 0.1% trifluoroacetic acid (TFA) and 0.5% acetonitrile (ACN) and desalted by reverse-phase tC18 Sep-Pak extraction cartridges (Waters Corporation, Milford, MA, USA), as reported previously with minor improvement [41]. Sep-Pak cartridges were conditioned by sequential washing with methanol, acetonitrile and 0.1% TFA and 0.5% ACN prior to loading of the peptide mixtures. Gravity was used for washing and loading of the samples. Peptides were eluted from Sep-Pak with 600 μL 90% ACN and 0.5% acetic acid 2 times. These two elutes were combined together for lyophilizing and further analysis.

### 3.4. An Off-Line High-pH HPLC Separation

Desalted peptide mixtures were re-suspended in 400 μL Buffer A (5% ACN, 10 mM ammonium formate, pH = 10) and fractionated using a Bonna-Agela C18 3 μm, 4.6 × 250 mm column on a RIGOL-L3000 HPLC (RIGOL, Beijing, China). For initial experiments, the HPLC was equilibrated by Buffer B (90% ACN, 10 mM ammonium formate, pH = 10) and Buffer A sequentially. Samples were loaded onto the column at 1 mL/min for 25 min, after which the fractionation gradient commenced as follows: 2% Buffer B to 10% Buffer B in 5 min, 10% to 27% Buffer B in 32 min, 27% to 31% Buffer B in 3 min, 31% to 39% Buffer B in 4 min, 39% to 50% Buffer B in 11 min, 50% to 80% Buffer B in 5 min. The chromatogram was recorded at 214 nm. The total number of fractions was set to 13 fractions, as the work flow described (Figure 1). All of these 13 fractions were lyophilized respectively. The dried peptide mixtures were used for further IMAC enrichment.

### 3.5. IMAC Enrichment

For the IMAC enrichment, a nickel-nitrilotriacetic acid (Ni-NTA) magnetic agarose bead slurry (IMAC beads, Qiagen, Dusseldorf, Germany) was used as previously described with minor modification [42]. Firstly, 10 μL Ni-NTA magnetic agarose bead slurry were centrifuged, and the supernatant was discard. Then, this was washed 3 times with 20 μL H_2_O. Twenty microliters of 100 mM EDTA, pH = 8.0, were used to treat the Ni-NTA magnetic beads for 1 h end-over-end. After removing the EDTA solution, beads were washed 3 times with water and then treated with 10 mM FeCl_3_ for another 1 h. After removing excess FeCl_3_, the beads were washed 3 times with water and resuspended in CH_3_OH:ACN:0.01% HAc = 1:1:1. Beads were then conditioned by loading buffer (80% ACN with 0.1% TFA) 3 times, followed by incubation with peptide mixtures (in 20 μL 80% ACN with 0.1% TFA) for 1 h. The phosphopeptides were eluted with 20 μL elution buffer (ACN:0.05% NH_4_OH = 1:1). The eluted phosphopeptides were immediately acidified with 5% FA and lyophilized for LC–MS/MS analysis.

### 3.6. Mass Spectrometric Analysis and Data Analysis

All phosphopeptide samples were analyzed on an LTQ Orbitrap Mass Spectrometer (Thermo Fisher Scientific, Waltham, MA, USA) equipped with a nanoAcquity Ultra Performance LC (UPLC) system (Waters Corporation, Milford, MA, USA), and all MS data were analyzed using the MaxQuant software suite (version 1.5.0.25, Martinsried, Germany). The HCD (High energy Collision Dissociation) MS/MS spectra were searched against an *in silico* tryptic digest of human proteins from the NCBI database (April, 2014). All MS/MS spectra were searched with the following MaxQuant parameters for phosphopeptide identification: precursor ion peaks were searched with an initial mass tolerance of 20 ppm; up to 2 missed cleavages were allowed, and peptides with at least six amino acids were retained; oxidation (+15.9949 Da) of methionine and phosphor (STY) was set as the variable modifications; cysteine carbamidomethylation was set as the fixed modification; peptide spectrum matches, proteins and sites were automatically filtered to a 1% false discovery rate based on the Andromeda score, peptide length and individual peptide mass errors.

### 3.7. Phosphorylation Motif Analysis

Motif-x [15] was adapted for the identification of the phosphorylation motifs present in the MHCC97-H cell line phosphoproteome data. We extracted all items of PSP (7, 7), a phosphopeptide with a length of 15 with 7 upstream residues and 7 downstream residues surrounding the p-site. If the p-site were located in the *N*-terminus or *C*-terminus of the protein sequence, the phosphopeptide was complemented to PSP (7, 7) with the necessary number of “*”s instead of any amino acid. Motif-x parameter settings of “pre-aligned,” central S, T or Y, width = 15, occurrence = 20 and significance = 0.000001 were adopted. Because of the upload restrictions, the inherent IPI *Human* proteome in Motif-x was set as the background.

### 3.8. Group-Based Prediction System (GPS) Algorithm with the Interaction Filter, or in Vivo GPS (iGPS) Analysis

All PSP (7, 7) items were prepared in the FATSA format. We used iGPS 1.0 [20] to predict the site-specific kinase-substrate relations for all identified phosphorylation proteins in our dataset. During the prediction, the medium threshold and the Exp./String interaction were chosen.

### 3.9. Analysis of Secondary Structures

For the analysis of the structure preferences for the p-sites, NetSurfP ver. 1.1 [25] with the default parameters was adapted for the prediction of the secondary structures.

### 3.10. The Functional Enrichment Analysis of the GO (Gene Ontology)/KEGG (the Kyoto Encyclopedia of Genes and Genomes) Pathway

For the functional enrichment analyses of the liver phosphorylation proteins identified in the MHCC97-H cell line, we downloaded the gene association files of GO (1 October 2014) from the database of the Gene Ontology Consortium (http://geneontology.org/) [43]. By mapping the phosphorylation proteins to the GO annotations, there were 42,961 UniProt proteins, including 2102 substrates in MHCC97-H annotated by at least one GO term. Furthermore, we purchased the FTP (File Transfer Protocol) subscription of KEGG for private use on 9 March 2012 [44]. A total of 6360 UniProt sequences with 399 substrates in MHCC97-H were annotated by at least one KEGG pathway. The hypergeometric test [45] was used to statistically analyze the enrichment functional distributions and pathways of the identified phosphorylation proteins.

## 4. Conclusions

Comprehensive analysis of the phosphoproteome on tumor cell lines can help us to understand the cascade of the signaling pathways mediated by phosphorylation in cancers. For example, Zhang *et al.*, had revealed novel phosphorylation events in insulin signaling regulated by protein phosphatase 1 regulatory subunit 12A by quantitative phosphoproteomics [46]. Although there were various published methods for phosphopeptide enrichment [47,48,49], we attempted to establish a new strategy to study the phosphoproteome of cell lines. In this study, we combined an off-line high pH HPLC separation strategy with multi-step IMAC to study the phosphoproteome on a human metastatic HCC cell line, MHCC97-H. After three rounds of IMAC enrichment on thirteen fractions, 6593 phosphopeptides of 2930 phosphoproteins were identified in the MHCC97-H cell line. By comparison of three rounds of IMAC steps, we found that one round of IMAC enrichment can cover almost all phosphopeptides from 2.5 mg of starting lysates. However, with respect to the ratio of phosphopeptides with more p-sites, the basic or hydrophobic features could be increased by more rounds of IMAC step.

Through analyzing GO categories of identified phosphoproteins by our method, we found that several transcription-related processes, such as transcriptional regulation, mRNA processing and RNA splicing, were over-presented in the MHCC97-H cell line. The enrichment analysis of the KEGG pathway indicated that the spliceosome pathway was the most significantly enriched pathway. Compared with a published phosphoproteome of human normal liver, we found that a number of proteins in the spliceosome pathway, such as U2AF2, EIF4A3, CDC5L and SMNDC1, were identified as phosphoproteins only in MHCC97-H. All of these results indicated that phosphorylation of spliceosome-related proteins may participate in HCC metastasis. Moreover, the phosphopeptide enrichment strategy established in this study may have identified novel phosphoproteins involved in liver cancer.

## Supplementary Materials

Supplementary materials can be found at http://www.mdpi.com/1422-0067/16/02/4209/s1.
